# Oridonin-Loaded Polyvinyl Alcohol/Chitosan Composite Films and Coatings: Characterization, Antibacterial Activity, and Preservation Efficacy on Chilled Chicken Breast

**DOI:** 10.3390/foods15203756

**Published:** 2026-10-21

**Authors:** Lunji Wang, Lintong Yang, Jiayi Zhao, Huijuan Li, Min Jiu

**Affiliations:** College of Food and Bioengineering, Henan University of Science and Technology, Luoyang 471023, China; lunjiwang@haust.edu.cn (L.W.); 250320070804@stu.haust.edu.cn (L.Y.); zhengyidehuoban111@163.com (J.Z.); 220320070736@stu.haust.edu.cn (H.L.)

**Keywords:** chitosan, oridonin, composite film, preservation, chilled chicken breast

## Abstract

Microbial spoilage significantly limits meat shelf life, necessitating degradable packaging with strong antibacterial properties. Oridonin (ORI), effective against foodborne pathogens, was incorporated into a polyvinyl alcohol (PVA)/chitosan (CS) matrix. This study examined ORI’s effects on the physical properties and antibacterial activity of the films and evaluated their preservation efficacy on chilled chicken breasts. Structural analysis suggested good compatibility of the composite films, likely ascribed to the H-bonding between ORI and the PVA/CS matrix. Increasing ORI content enhanced mechanical performance, reduced surface wettability, and improved light barrier properties, while also significantly improving antibacterial capacity. The composite film containing 75 μg/mL ORI exhibited optimal overall performance and potent antibacterial activity against *Staphylococcus aureus*, *Listeria monocytogenes*, and *Escherichia coli*, as evidenced by inhibition zone diameters of 19.51, 18.64, and 16.36 mm, respectively. Preservation experiments showed that film and coating containing 75 μg/mL ORI significantly inhibited the rise in TVB-N concentration, pH, total bacterial count, and juice loss rate of chilled chicken breast during storage. The coating generally showed slightly better preservation performance than the pre-formed film and extended the shelf life of chilled chicken breast by 4 days. This study provided a foundation for PVA/CS@ORI films and coatings as antimicrobial packaging for chilled chicken breast.

## 1. Introduction

Chicken meat is widely favored by consumers due to its nutritional benefits, including low calorie content, minimal fat, and reduced cholesterol levels. Compared to beef and pork, chicken lipids contain a greater proportion of unsaturated fatty acids, which aid in lowering low-density lipoprotein (LDL) cholesterol—a significant cardiovascular disease risk factor. Additionally, chicken meat boasts excellent protein content with great digestibility and a complete amino acid profile for efficient absorption and utilization [1]. However, preserving chilled chicken presents significant challenges. Exudate accumulation during storage not only compromises product quality but also creates favorable conditions for microbial proliferation. Microorganisms originating from either the meat itself or the production, storage, and transportation environments can cause undesirable effects, such as off-flavors, surface sliminess, and nutrient degradation [2]. Meanwhile, lipid oxidation leads to color alterations, while myoglobin contributes to meat discoloration [3]. Enzymatic activity also negatively impacts meat sensory attributes, including flavor, texture, and visual appearance [4]. Effective packaging solutions are therefore essential to mitigate these degradation pathways, preserve freshness, increase shelf life, and provide food safety.

Food packaging preserves food quality and nutritional value over extended periods by protecting contents from external physical, chemical, and biological factors. With increasing public awareness and demand for food quality and safety, developing efficient, eco-friendly, highly antimicrobial, and non-toxic food packaging has become a key research focus. Green coating materials are primarily divided into two categories: edible types, such as polysaccharides and proteins, and non-edible types, such as polyvinyl alcohol (PVA) [5]. Chitosan (CS), obtained through deacetylation from abundant crab shell resources, is favored for its biocompatible, biodegradable, renewable, antimicrobial, and filmogenic characteristics. These attributes make it an exceptional polysaccharide-based coating material [6,7]. Currently, CS is used extensively in food, fruit, and vegetable packaging, including preservation of litchi [8], strawberry [9,10], yellow cherry tomato [11], beef steak [12,13], and so on. However, single-component chitosan films have the disadvantages of poor water resistance and low mechanical strength, and thus are often blended with other polymers to expand their application range [14].

PVA, a biodegradable and non-toxic synthetic polymer, has attracted significant attention because of its exceptional miscibility, high barrier properties, transparency, toughness, biocompatibility, and film-forming capabilities [15]. PVA films show high mechanical strength, chemical stability, and excellent tolerance to both alkaline and acidic media. The United States Department of Agriculture has authorized the use of PVA films in meat product packaging [16]. However, pure PVA materials show notable deficiencies in hydrophobic properties and antimicrobial functionality. Since CS molecules contain hydroxyl and amino reactive groups, they can form a compatible system with PVA through hydrogen bonding interactions [17]. PVA/CS composite films not only display outstanding biodegradability and biocompatibility but also effectively address the issue of PVA’s high water solubility. Recent studies have increasingly explored PVA/CS-based active food materials to improve their barrier and mechanical characteristics and functionalize them with natural bioactive agents such as essential oils and polyphenols, resulting in enhanced antioxidant or antimicrobial activity in films and extended food shelf life [18,19].

Antibacterial active substances derived from plants offer natural safety and are easily degradable in the natural environment. In recent years, a wide range of natural antimicrobial substances, including essential oils, polyphenols, and plant extracts, has been incorporated into active food packaging to improve food safety and extend shelf life [20,21]. For example, cinnamaldehyde-loaded chitosan nanoparticles incorporated into polysaccharide films effectively inhibited foodborne pathogens and extended the shelf life of meat products [22]. Oridonin (ORI), the primary bioactive ent-kaurane diterpenoid in *Rabdosia rubescens* (traditional Chinese herbal medicine Donglingcao), possesses potent antitumor, antibacterial, antioxidant, and anti-inflammatory effects. Prior reports have confirmed that ORI can suppress the growth of foodborne pathogens, including *Aeromonas hydrophila*, *Vibrio parahaemolyticus*, *L. monocytogenes*, and *S. aureus* [23,24,25]. In China, the leaves of *R. rubescens* are harvested, processed, and dried to make an herbal tea that has been used for the prevention of illness and the enhancement of health. However, to the best of our knowledge, studies on ORI-loaded active food packaging films or edible coatings remain scarce. Therefore, this study developed novel PVA/CS hybrid films with antibacterial activity via the incorporation of varying ORI concentrations, and investigated the influence of ORI on their physicochemical, mechanical, microstructural, and antibacterial properties. Their preservation efficacy was further validated on chilled chicken breast, providing new perspectives for biodegradable antimicrobial packaging design.

## 2. Materials and Methods

### 2.1. Reagents

Oridonin (CAS 28957-04-2; HPLC purity > 98%) was obtained from Shanghai Yuanye Bio-Technology Co., Ltd. (Shanghai, China), and made into a stock solution by dissolving it in ethanol. Chitosan (CS, CAS 9012-76-4, 95% deacetylated, MW = 30,000) and Polyvinyl alcohol (PVA, weight-average molar mass: 0.9–1.5 × 10^4^ g/mol, type: 2488) were supplied by Shanghai Macklin Biochemical Co., Ltd (Shanghai, China). The remaining chemical reagents were all of analytical quality and obtained from Sinopharm Chemical Reagent Co., Ltd. (Shanghai, China), unless otherwise indicated. Fresh chilled chicken breasts and polyethylene (PE) films were obtained from a local supermarket (Dazhang, Luoyang, China). *Staphylococcus aureus* ATCC6538, *Listeria monocytogenes* ATCC19115, and *Escherichia coli* ATCC25922 were acquired from the China Center for Type Culture Collection and kept at a temperature of −80 °C in a nutrient broth (NB, Qingdao Hope Bio-Technology Co., Ltd., Qingdao, China) containing 200 mL/L glycerol.

### 2.2. Synthesis of PVA/CS@ORI Composite Films

The solution casting approach was employed to prepare PVA/CS@ORI composite films. CS (0.54 g) was dissolved in 100 mL of 10 mL/L acetic acid solution under magnetic stirring for 10 h. PVA (10.85 g) was dissolved in 100 mL of deionized water at 80 °C under magnetic stirring for 8 h. The CS and PVA solutions were then mixed at a 4.8:5.2 volume ratio, followed by the addition of glycerol with a final concentration of 9.7 g/L as a plasticizer. Pipette 0, 50, 100, 150, and 200 μL of ORI stock solution (5 mg/mL), respectively, and add each to 10 mL of the PVA/CS mixture to obtain film-forming solutions with final ORI concentrations of 0, 25, 50, 75, and 100 μg/mL. The film-forming solution (10 mL) was ultrasonicated (KQ3200DV, Kunshan Ultrasonic Instruments Co., Ltd., Kunshan, China; 40 W, 40 °C, 20 min) and poured into 9 cm diameter glass Petri dishes. The resulting PVA/CS@ORI composite films were oven-dried for 10 h at 45 °C (DHG-9246A, Shanghai Jinghong Laboratory Instrument Co., Ltd., Shanghai, China). The nominal ORI contents in the dried films were approximately 3.93, 7.86, 11.79, and 15.72 μg/cm^2^ of film area for the 25, 50, 75, and 100 μg/mL formulations, respectively. PVA/CS films without ORI, used as controls, were prepared using the same method. After peeling from the casting plates, all film samples were conditioned in a glass desiccator for 48 h at 25 °C and 50% relative humidity (RH) prior to further use.

### 2.3. Analysis of Physical Characteristics of Composite Films

#### 2.3.1. Film Thickness and Transparency

Thickness was gauged at ten random sites with a precision micrometer (0.001 mm; Dongguan Jingyou Mold Hardware Co., Ltd., Dongguan, China), and optical transmittance was measured by UV-Vis spectrophotometry (UVI800PC, Shanghai Jinghua Technology Instrument Co., Ltd., Shanghai, China).

#### 2.3.2. Mechanical Characteristics

Elongation at break (EAB) and tensile strength (TS) were determined following a technique modified from Liu et al. (2024) [26]. The specimens were conditioned inside a desiccator at 25 °C and a RH of 55% ± 5% for 24 h after being cut into 80 mm × 10 mm strips. Mechanical characteristics were analyzed at least five times via a texture analyzer (Instron5544, Instron, Norwood, MA, USA) with a 50 mm/min tensile rate and determined via Equations (1) and (2):

(1) TS/MPa=F/L×W

(2) $$EAB/\%=\left(L_{1}-L_{0}\right)/L_{0}$$

where *F* denotes the maximum load (N), and *L*, *W*, $L_{1}$, and $L_{0}$ indicate the film width, film thickness, the stretched length at break, and the initial film length, all in mm, respectively.

#### 2.3.3. Moisture Content (MC), Water Absorption (WA), and Water Solubility (WS)

MC, WA, and WS were measured following established methods [27,28] with minor modifications. Film specimens (2 cm × 2 cm) were weighed ($W_{0}$), dried at 50 °C for 24 h to obtain $W_{1}$, then immersed in 50 mL DI water at ambient temperature for 24 h under stirring. After surface drying with absorbent paper, saturated weights ($W_{t}$) were recorded, followed by drying at 60 °C to constant weight ($W_{d}$). The values of MC, WS, and WA were determined separately with Equations (3)–(5).

(3) $$MC/\%=\left(W_{0}-W_{1}\right)/W_{0}\times 100$$

(4) $$WA/\%=\left(W_{t}-W_{1}\right)/W_{1}\times 100$$

(5) $$WS/\%=\left(W_{1}-W_{d}\right)/W_{1}\times 100$$

#### 2.3.4. Water Vapor Permeability (WVP)

WVP was recorded via a method adapted from Yuan et al. (2024) [29]. Samples were sealed in fabric moisture permeability cups (YG-11, Hangzhou Boyn Instrument Co., Ltd., Hangzhou, China) containing 35 g of completely dried calcium chloride. These cups were placed in desiccators equilibrated at 50% RH and 23 °C using saturated Mg(NO_3_)_2_ solutions. WVP values, calculated from four replicates per sample, were calculated using Equation (6):

(6) $$WVP=\frac{∆m\times d}{A\times t\times ∆p}$$

where Δ*m* (g) denotes the difference in mass between two consecutive readings over time, *A* (m^2^) indicates the test area (cup mouth area), *d* (mm) represents the film thickness, Δ*p* (Pa) represents the pressure difference across the film (1405.2 Pa), and *t* (s) denotes the time interval between measurements. The units of WVP are g·mm·m^−2^·s^−1^·Pa^−1^.

#### 2.3.5. Water Contact Angle (WCA)

WCA was evaluated following Yuan et al.’s (2024) [29] method. Film samples mounted on glass slides were deposited with deionized water droplets (3 µL) and equilibrated for 10 s. Measurements were conducted in triplicate at room temperature by a contact angle (CA) goniometer (OCA15EC, LAUDA Scientific, Lauda-Königshofen, Germany), with angles automatically captured by integrated SCA20 software based on digital image analysis.

### 2.4. Analysis of Interaction Between Components

#### 2.4.1. Fourier Transform Infrared Spectroscopy (FTIR) Analysis

The molecular structures and intermolecular interactions of the films were analyzed using FTIR spectroscopy (Vertex70, Bruker Optik GmbH, Ettlingen, Germany; 4 cm^−1^ resolution, 4000–400 cm^−1^ range). Measurements employed a 6 mm aperture, 10 kHz scanner velocity, with 32 background/sample scans at 64 accumulations. Spectra were recorded in absorbance mode versus wavenumber.

#### 2.4.2. Thermal Stability

Thermal properties were characterized with simultaneous thermal analysis (STA) on a TGA/DSC 1 system (Mettler-Toledo GmbH, Nänikon, Switzerland). Briefly, thermogravimetric (TGA), thermogravimetry (DTG), and differential scanning calorimetry (DSC) data were acquired simultaneously. For DSC, 7 mg samples in alumina crucibles were heated from 25 to 500 °C at a ramp rate of 10 °C/min. TGA was performed under identical heating conditions using a thermobalance. Both analyses were conducted under inert gas (50 mL/min) to prevent oxidative interference.

#### 2.4.3. X-Ray Diffraction (XRD) Analysis

The crystalline structure was examined by XRD using a Bruker D8 Advance diffractometer (Cu Kα radiation, Bruker AXS, Karlsruhe, Germany). Diffraction patterns were captured within a 2*θ* range spanning from 5° to 80° at a scan rate equal to 2°/min, with 40 kV and 40 mA instrument operating conditions [30].

#### 2.4.4. Morphology Studies

The cross-sectional and surface morphologies were assessed via a scanning electron microscope (SEM) (TM3030Plus, Hitachi High-Tech Corporation, Tokyo, Japan) fitted with a highly sensitive low-vacuum secondary electron detector. Before imaging, samples were sputter-coated with a thin gold layer after being cryo-fractured in liquid nitrogen to reduce charging. Imaging was conducted at 10 kV.

### 2.5. Antibacterial Activity

Antibacterial activity was assessed by the Oxford cup method. Strains were inoculated into NB and incubated in a temperature-controlled shaker (Boxun, Shanghai, China) for 24 h at 37 °C with shaking (180 r/min). The cultures were then centrifuged at 6000 r/min for 10 min, and the cell pellets were washed and resuspended in sterile saline (8.5 g/L NaCl, Sinopharm Chemical Reagent Co., Ltd., Shanghai, China). A total of 10 mL nutrient agar (NA, Qingdao Hope Bio-Technology Co., Ltd., Qingdao, China) was added into sterile Petri dishes and allowed to cool and solidify, followed by placement of five sterile Oxford cups in a defined arrangement. Next, 0.1 mL bacterial suspension (~1 × 10^8^ CFU/mL, 0.5 McFarland turbidity) was fully blended with 100 mL NA (~46 °C), and the mixture (10 mL) was poured into a sterile plate. After solidification, the Oxford cups were removed with sterile forceps. Then, aliquots (100 μL) of the original film-forming solutions (PVA/CS or PVA/CS@ORI) were individually loaded into the wells and subjected to 24 h incubation at 37 °C, followed by quantification of antimicrobial activity by measuring inhibition zone diameters. All manipulations were performed in a laminar flow hood. The antibacterial activity was classified according to reported criteria: zone diameter ≥ 17 mm, strong activity; 12–16 mm, moderate activity; 7–11 mm, weak activity [31].

### 2.6. Application in Chicken Breast Preservation

#### 2.6.1. Chicken Breast Treatment and Storage

Under sterile conditions, the chicken breasts were washed with sterile water and cut into uniformly sized portions (approximately 3 × 2 × 2 cm). Subsequently, the meat samples (three replicates were prepared at each sampling point, with each replicate representing an independent sample unit) were randomly divided into five groups: chicken fillets wrapped in PE films (control, CK), those packaged with PVA/CS film without ORI (PVA/CSF), packaged in PVA/CS film incorporated with 75 μg/mL ORI (PVA/CS@ORIF), packaged with PVA/CS film-forming solution without ORI (PVA/CSFS), packaged in PVA/CS film-forming solution incorporated with 75 μg/mL ORI (PVA/CS@ORIFS). Two application modes were evaluated in this study. For the PE, PVA/CSF, and PVA/CS@ORIF film packaging groups, chicken pieces were placed in the center of the films, and the films were folded over the samples to completely cover the surface to form a pouch-like package. For the PVA/CSFS and PVA/CS@ORIFS coating groups, chicken samples were dipped in the designated solutions for 30 s, air-dried inside a laminar flow hood for a duration equal to 30 min. The five sample groups were then stored at 4 °C. TVC, pH, TVB-N, and TBARS values were measured at 2-day intervals over a 10-day storage period.

#### 2.6.2. pH

Changes in chicken breast pH were examined using a modified approach based on Kanatt (2020) [32]. In brief, samples (5 g) were homogenized for 5 min with distilled water (50 mL), followed by pH measurement utilizing a PHS-3C digital pH meter (INESA Scientific Instrument Co., Ltd., Shanghai, China).

#### 2.6.3. Mass Loss Rate

After measurement of the initial masses ($m_{1}$) across the five treatment groups, the specimens were individually packed in resealable bags and refrigerated at a temperature equal to 4 °C. Every two days, after blotting surface exudates with filter paper, the sample mass ($m_{2}$) was re-measured. The mass loss rate was derived from Equation (7) [33]:

(7) $$Mass\;loss\;rate/\%=\left(m_{1}-m_{2}\right)/m_{1}\times 100$$

#### 2.6.4. Thiobarbituric Acid Reactive Substances (TBARS)

The TBARS were assessed following Cai et al. (2021) [34] and stated as mg of malonaldehyde (MDA)/kg of meat [32]. Briefly, homogenization of 5 g specimens with trichloroacetic acid (25 mL, 7.5 g/L) was carried out for 30 min under shaking. Following filtration, supernatant (5 mL) was combined with thiobarbituric acid (TBA, 0.83 mg/mL, 5 mL), heated within a boiling water bath for a duration equal to 40 min, cooled, and subsequently subjected to centrifugation for a duration equaling 10 min at a rate equal to 8000 r/min. TBARS were calculated from a standard curve of 1, 1, 3, 3-tetraethoxypropane at 532 nm using Equation (8) [33].

(8) $$TBARS(mg/kg)=A_{532}/m\times 9.48$$

Here, $A_{532}$ is the specimen absorbance at 532 nm, and *m* is its mass (g).

#### 2.6.5. Total Volatile Basic Nitrogen (TVB-N)

TVB-N (mg N/100 g) was determined according to the Chinese National Standard GB 5009.228–2016 [35]. In brief, samples (10 g) were homogenized by shaking for 30 min with distilled water (50 mL). Following filtration, the filtrate (10 mL) was mixed with MgO suspension (5 mL, 10 g/L) in a reaction chamber, which was immediately sealed. Following steam distillation (5 min), the distillate was captured in 10 mL of boric acid solution (20 g/L). Finally, as indicators, methyl red and methylene blue were used to titrate the mixture with 0.01 mol/L HCl.

#### 2.6.6. Color

The color of chilled chicken breast was analyzed utilizing a modified version of Nandi & Guha (2024) [36]. Chromatic parameters (b*: yellowness, a*: redness, and L*: lightness) were recorded with a colorimeter (color i5, X-Rite Incorporated, Grand Rapids, MI, USA).

#### 2.6.7. Total Viable Count (TVC)

TVC of chilled chicken breast was determined following the Chinese National Standard GB 4789.2-2022 [37]. Briefly, 25 g of samples were minced, soaked in sterile saline (225 mL), followed by 2 min of homogenization at 10,000 r/min. Plate count agar (PCA, Qingdao Hope Bio-Technology Co., Ltd., Qingdao, China) was used to plate the appropriate dilutions after serial dilutions were made. Following incubation for 48 h at a temperature equal to 37 °C, viable colonies were enumerated to determine TVC.

### 2.7. Statistical Analysis

The experimental data were expressed as mean ± standard deviation (SD). Statistical analyses were carried out via SPSS 24.0 (SPSS Inc., Chicago, IL, USA). One-way ANOVA followed by Duncan’s multiple range test was employed to examine significant differences between groups at *p* < 0.05. Graphical representations were prepared via Origin 2022 software (OriginLab, Northampton, MA, USA).

## 3. Results and Discussions

### 3.1. Physicochemical Properties of Films

#### 3.1.1. Film Thickness and Optical Properties

Thickness values of PVA/CS@ORI films are summarized in Table 1. No statistically notable variations were observed between samples regardless of ORI addition (*p* > 0.05). Film thickness is influenced by various factors, such as fabrication method, drying parameters, solution dosage, and film formulation [38]. Here, all films were fabricated via solution casting using identical volumes of the PVA/CS@ORI casting solution on substrates with the same surface area, so their thicknesses were nearly uniform. Therefore, the addition of ORI at different mass fractions does not influence film thickness. The optical characteristics of packaging films greatly affect their appearance and consumer preference [29]. Table 1 shows that the light transmittance of the PVA/CS film reached 95.73% at 600 nm. In contrast, the PVA/CS@ORI films displayed a significant reduction in transmittance (from 94.67% to 90.87%) as the ORI content increased (*p* < 0.05). This marked decrease indicates that ORI incorporation effectively imparts light-blocking capacity to the films, which helps inhibit photodegradation and thus enhances the shelf stability of packaged products.

#### 3.1.2. Mechanical Properties

Mechanical testing of PVA/CS@ORI films was performed to ascertain their food packaging feasibility. These parameters critically determine the composite’s capacity to maintain dimensional stability and withstand mechanical stresses during storage and transportation [39]. Table 1 reveals that the inclusion of ORI significantly improved the mechanical behavior of the films. The neat PVA/CS film showed baseline TS and EAB values of 26.15 MPa and 105.13%, respectively. With ORI concentrations ranging from 25 to 75 μg/mL, both TS and EAB showed progressive increases, reaching peak values of 31.30 MPa and 165.88%, respectively, at 75 μg/mL. This enhancement was ascribed to the hydrogen-bonding interactions facilitated by ORI incorporation [40,41]. At the highest concentration tested (100 μg/mL), however, both TS and EAB showed a decreasing trend. The decline in mechanical properties may result from excessive ORI addition, which disrupted the original system structure.

#### 3.1.3. Hydrophobicity, Moisture and Water Solubility

**Figure 1:**
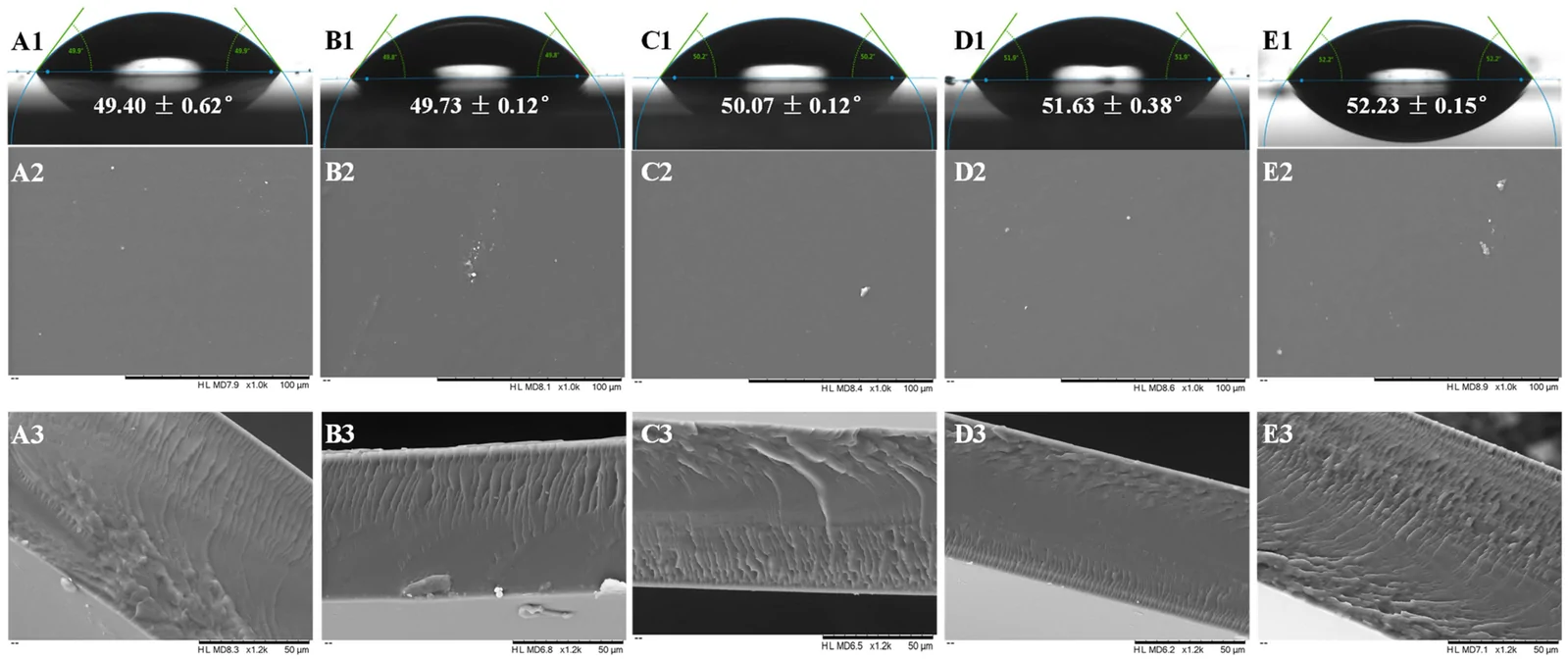
The water contact angle (**A1**–**E1**), surface ((**A2**–**E2**), 1000×) and section ((**A3**–**E3**), 1200×) SEM images of composite film. Notes: A-E are PVA/CS film, 25 µg/mL, 50 µg/mL, 75 µg/mL and 100 µg/mL PVA/CS@ORI composite films, respectively.

As a critical interfacial characteristic governing liquid–solid interactions, wettability serves as a pivotal determinant in evaluating the surface hydrophilicity/hydrophobicity balance of packaging substrates. The strategic optimization of surface hydrophobic attributes emerges as a vital prerequisite for ensuring long-term functional integrity in advanced packaging systems. This surface property not only exhibits profound correlations with essential functional parameters encompassing gas barrier performance, microbial inhibition capability, and chemical resistance stability, but also closely governs the spreading behavior of liquids on the material surface [42]. Static water CA measurements were employed to evaluate the water barrier property of PVA/CS@ORI films (Figure 1(A1–E1)). PVA/CS film exhibited a CA of 49.40 ± 0.62°, indicative of its hydrophilic nature, which is attributable to the abundant hydrophilic functional groups present in both CS and PVA [43,44]. Upon ORI incorporation, the surface wettability decreased progressively, as reflected by the increasing CA values from 49.73 ± 0.12° to 52.23 ± 0.15°. Although all films remained hydrophilic (CA < 90°), the increase in CA indicates a relative enhancement of surface hydrophobicity.

WA and WS, key indicators of moisture sensitivity, are summarized in Table 1. Moisture content showed no significant differences across groups (*p* > 0.05), while both WA and WS decreased with increasing ORI content. This can be linked to the good hydrophilicity of CS and PVA, whereas ORI is not water-soluble. The presence of ORI affected the water absorption rate and diffusion coefficient. Consequently, as ORI content increased from 25 to 100 μg/mL, the WA of the PVA/CS@ORI films decreased significantly from 995.89% to 625.33% (*p* < 0.05), while the WS gradually declined from 91.75% to 88.57%. The reduction in WA and WS likely results from the enhanced hydrophobicity of the films at higher ORI concentrations, as hydrophobic ORI molecules filled the original pores and created a denser film structure, thereby partially limiting water absorption and dissolution [45]. Previous studies have demonstrated that essential oil incorporation significantly reduces both WA and WS in film systems [46,47]. Similarly, Rhim (2011) [48] reported that clay played a vital role in forming nanocomposite films, decreasing water solubility with increasing nanoclay content, an effect ascribed to the tortuous diffusion pathways for water vapor and the strong interfacial interactions between the polymer matrix and intercalated silicate layers. In the present case, the observed reduction in WA and WS may be similarly explained by the interactions between the oxygen-containing moieties of ORI (e.g., -OH and -C=O groups) and the hydroxyl moieties of PVA and CS. Such intermolecular associations are believed to diminish the availability of free hydroxyl sites for water binding, thereby yielding a composite film with enhanced water resistance [49].

#### 3.1.4. Water Vapor Properties

WVP reflects a film’s ability to protect packaged contents from ambient moisture. Low WVP retards microbial proliferation and food deterioration by minimizing moisture transfer between the food product and the surrounding environment, thereby extending its shelf life. The data summarized in Table 1 confirm that the WVP of the PVA/CS@ORI films decreased with increasing ORI content, reaching a minimum of 11.32 ± 0.56 at 75 μg/mL ORI. This value was substantially lower compared to that of the PVA/CS film (*p* < 0.05). The reduction is attributable to the hydrophobic nature of ORI, which lowers the adsorption and diffusion rates of water molecules and enhances the fiber’s water vapor barrier. According to prior reports [50,51], hydrophobic lipid compounds (e.g., essential oils) in the film network reduce WVP, thereby limiting moisture transfer from the food to the environment.

### 3.2. Morphological and Structural Characterization of Films

#### 3.2.1. Film Microstructure

Film performance is intimately related to its microstructural attributes. Research indicates that increased structural density significantly improves both mechanical strength and barrier effectiveness in polymeric films [50]. As revealed by Figure 1(A2–E2), the inclusion of ORI into the PVA/CS matrix initially yielded films with outstanding surface uniformity and a smooth morphology, suggesting excellent ORI dispersion in the film-forming solution. However, progressive increases in ORI concentration led to noticeable surface irregularities and spot formation. Cross-sectional analysis reveals that PVA/CS@ORI composite films exhibit textured fracture surfaces with adhesive characteristics, consistent with ductile fracture behavior and indicative of enhanced tensile properties (Figure 1(A3–E3)). These morphological observations correlate well with mechanical testing data. Optimal film performance, characterized by superior component compatibility and formation quality, is achieved at an ORI concentration of 75 µg/mL.

#### 3.2.2. FTIR Analysis

**Figure 2:**
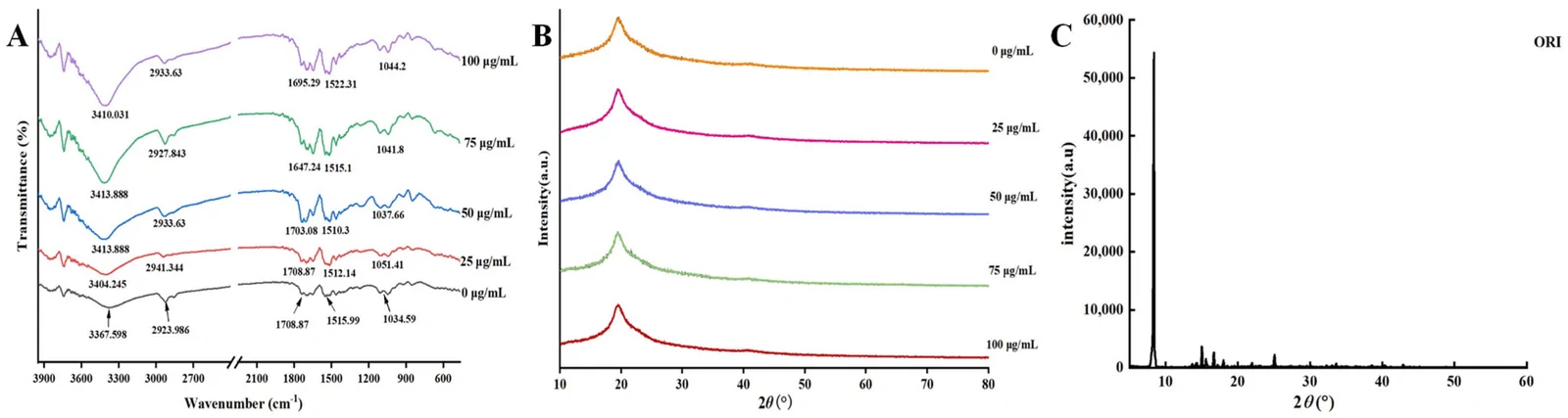
FTIR and XRD spectra of the composite films with different proportions of ORI. Notes: (**A**) FTIR spectra of the composite films, (**B**) XRD patterns of the composite films, (**C**) XRD pattern of ORI.

Possible intermolecular interactions between ORI and the PVA/CS matrix, along with the corresponding functional groups, were probed via FTIR spectroscopy, with the resulting data displayed in Figure 2A. For the PVA/CS film, the bands at approximately 3367.598 cm^−1^ were ascribed to O–H stretching vibrations and intermolecular hydrogen bonds arising from PVA–CS interactions [27]. Upon ORI incorporation, this band shifted to 3410.031 cm^–1^, accompanied by a marked increase in peak intensity with rising ORI concentration. This shift may suggest that the addition of ORI strengthened intermolecular interactions, possibly leading to a denser film network. The peak shift may result from hydrogen bond formation within the film matrix [38]. Additional characteristic bands were observed at 2923.986/2850.693 cm^−1^ (C–H and -CH_2_ of PVA and CS), 1652.939 (amide-I C=O), 1550.715 (amide-II N–H), 1461.993 (C–N), 1272.975 (amide-III N–H), 1110.960 (primary alcohol C–O), and 1045 cm^−1^ (glycerol O–H). Notably, comparison of the spectra revealed that the ORI-containing films exhibited no new bands, only changes in peak intensity, indicating that the overall structure remained unchanged and that all components were mutually compatible.

#### 3.2.3. XRD Analysis

The XRD pattern of PVA/CS film (Figure 2B) displayed a comparatively sharp diffraction peak at 19.5° (2*θ*), pointing to the presence of crystalline fractions. As reported in previous studies, chitosan exhibits a peak around 20°, corresponding to crystallization from its regular lattice structure [52]. PVA exhibits a diffraction peak at 19.6°, corresponding to its (101) crystal face [30,53]. Additionally, ORI exhibited characteristic crystallization peaks at 2*θ* = 8.4°, 13.6°, 14.2°, 15.1°, 15.6°, 16.9°, 17.9°, 22.5° and 25.1°, which indicates a complete crystalline structure of ORI (Figure 2C). Meanwhile, the XRD profiles of the ORI-loaded films showed no noticeable changes compared to those of the PVA/CS film, regardless of ORI concentration. This may be attributed to the low loading amount of ORI, reduced crystallinity, amorphization of ORI dispersed in the polymer matrix, small crystallite size, and possible overlap with the broad PVA/CS diffraction peak. Taken together, these findings demonstrate favorable compatibility among PVA, CS and ORI.

### 3.3. Thermal Characteristics

**Figure 3:**
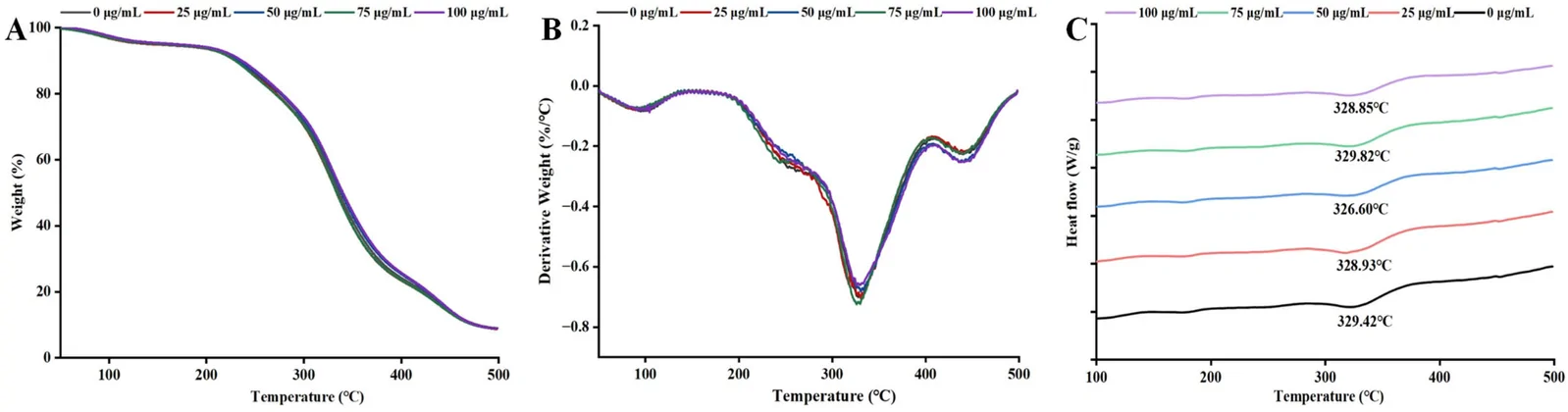
TG (**A**), DTG (**B**) and DSC (**C**) curves of the composite films with different proportions of ORI.

Thermal characteristics of the polymeric films were analyzed via simultaneous thermal analysis (STA). As illustrated in Figure 3, the TG, DTG, and DSC profiles of the PVA/CS@ORI films exhibited three distinct weight-loss stages across all formulations. The DSC thermograms (Figure 3C) revealed that these mass losses were accompanied by concurrent endothermic transitions. The first weight loss step, from 60 °C to 130 °C, corresponded to the dehydration of adsorbed and bound H_2_O molecules within the film samples [54]. The second weight loss step, occurring around 178 °C, corresponds to the decomposition of glycerin [55]. The final step is the rapid polymer decomposition stage between 200 °C and 500 °C, which involves the cleavage of the polymer backbone and the disruption of inter- and intramolecular hydrogen bonds [28,54]. The pyrolysis temperature profiles and weight loss rates of PVA/CS@ORI films were found to be comparable to those of blank PVA/CS films, indicating that varying the ORI content did not appreciably alter the thermal degradation behavior.

### 3.4. Antibacterial Performance

**Figure 4:**
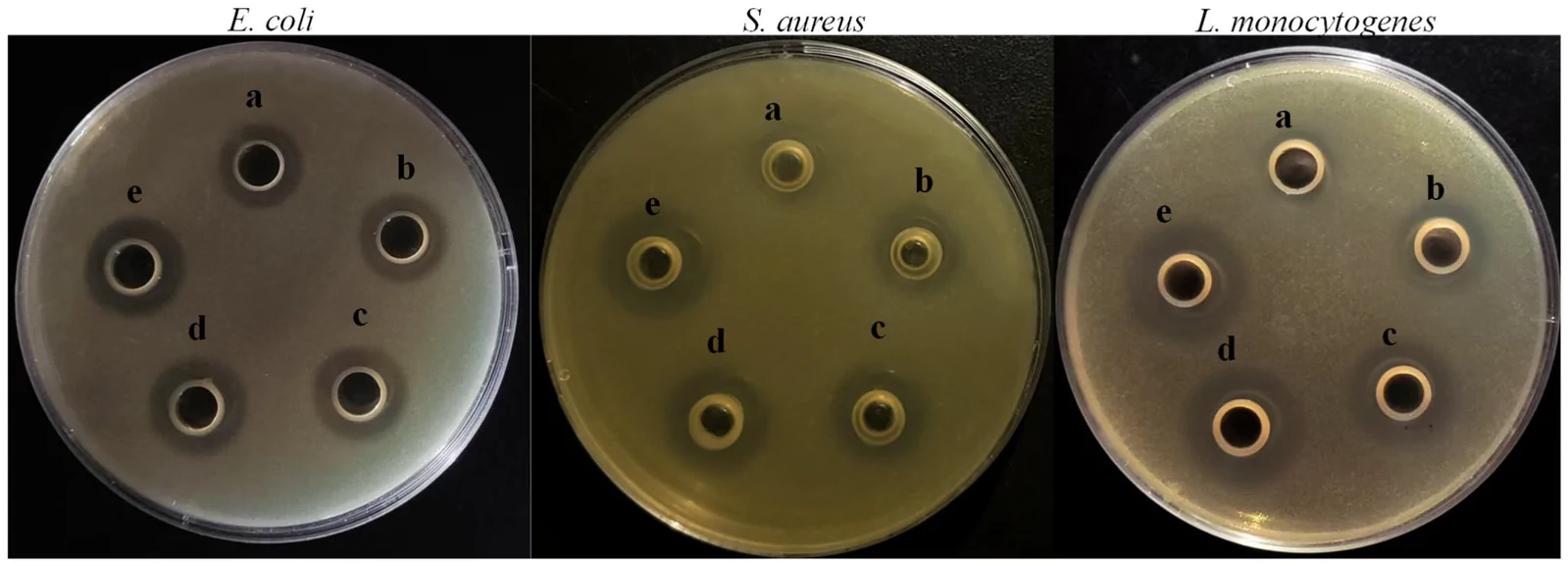
Antibacterial activity of the composite films against *E. coli, S. aureus* and *L. monocytogenes*. Notes: Oridonin concentration: a (0 μg/mL), b (25 μg/mL), c (50 μg/mL), d (75 μg/mL), e (100 μg/mL).

Effective inhibition of foodborne pathogenic bacteria in the contact environment is the primary condition for the effective application of composite membranes in food packaging. ORI-incorporated PVA/CS films were tested for antibacterial activity by the Oxford cup method. As illustrated in Figure 4 and Table 2, the PVA/CS film produced inhibition zones of 12.30 ± 0.04 mm (*S. aureus*), 12.27 ± 0.03 mm (*L. monocytogenes*), and 13.38 ± 0.03 mm (*E. coli*), corresponding to moderate antibacterial activity. This intrinsic activity is attributable to chitosan. In contrast, the incorporation of ORI markedly improved the antibacterial performance against all tested bacteria. At 75 μg/mL ORI, the inhibition zones reached 19.51 ± 0.05 mm (*S. aureus*), 18.64 ± 0.03 mm (*L. monocytogenes*), and 16.36 ± 0.04 mm (*E. coli*), with *S. aureus* and *L. monocytogenes* reaching the strong activity range. At 100 μg/mL ORI, the inhibition zones further increased to 20.47 ± 0.04 mm (*S. aureus*), 20.23 ± 0.05 mm (*L. monocytogenes*), and 17.98 ± 0.08 mm (*E. coli*), all within the strong activity range. The ORI-incorporated films consistently exhibited greater antibacterial efficacy against *S. aureus* and *L. monocytogenes* than against *E. coli*, and this differential effect intensified with increasing ORI concentrations. The observed disparity is likely due to the lipopolysaccharide envelope of *E. coli*, which functions as a physical barrier limiting ORI access to the bacterial cell. CS contains free amino groups, which, after being protonated, will interact with negatively charged microbial cell membranes, causing the cells to rupture or be damaged and thus achieving the bactericidal effect [56,57]. ORI inhibited bacteria by disrupting cell permeability, disrupting metabolism, and causing bacterial death [23,25]. Previous reports supported that the antimicrobial efficacy of chitosan films loaded with active agents (e.g., cinnamaldehyde, silver nanoparticles, gallic acid, lysozyme) has been enhanced compared to native chitosan [19,21,40,58]. Taken together, these results demonstrate a dose-dependent antibacterial effect of the ORI-loaded PVA/CS films, with higher ORI concentrations yielding greater inhibitory activity. This concentration-responsive behavior highlights their potential as active packaging materials to prevent bacterial contamination and retard food spoilage.

Although the highest antibacterial activity was observed at 100 μg/mL ORI, the composite film containing 75 μg/mL ORI was selected for the preservation experiment because it exhibited the best overall balance among mechanical strength, elongation at break, water vapor barrier property, and antibacterial activity.

### 3.5. Application in Chicken Breast Storage

#### 3.5.1. Color Value

**Figure 5:**
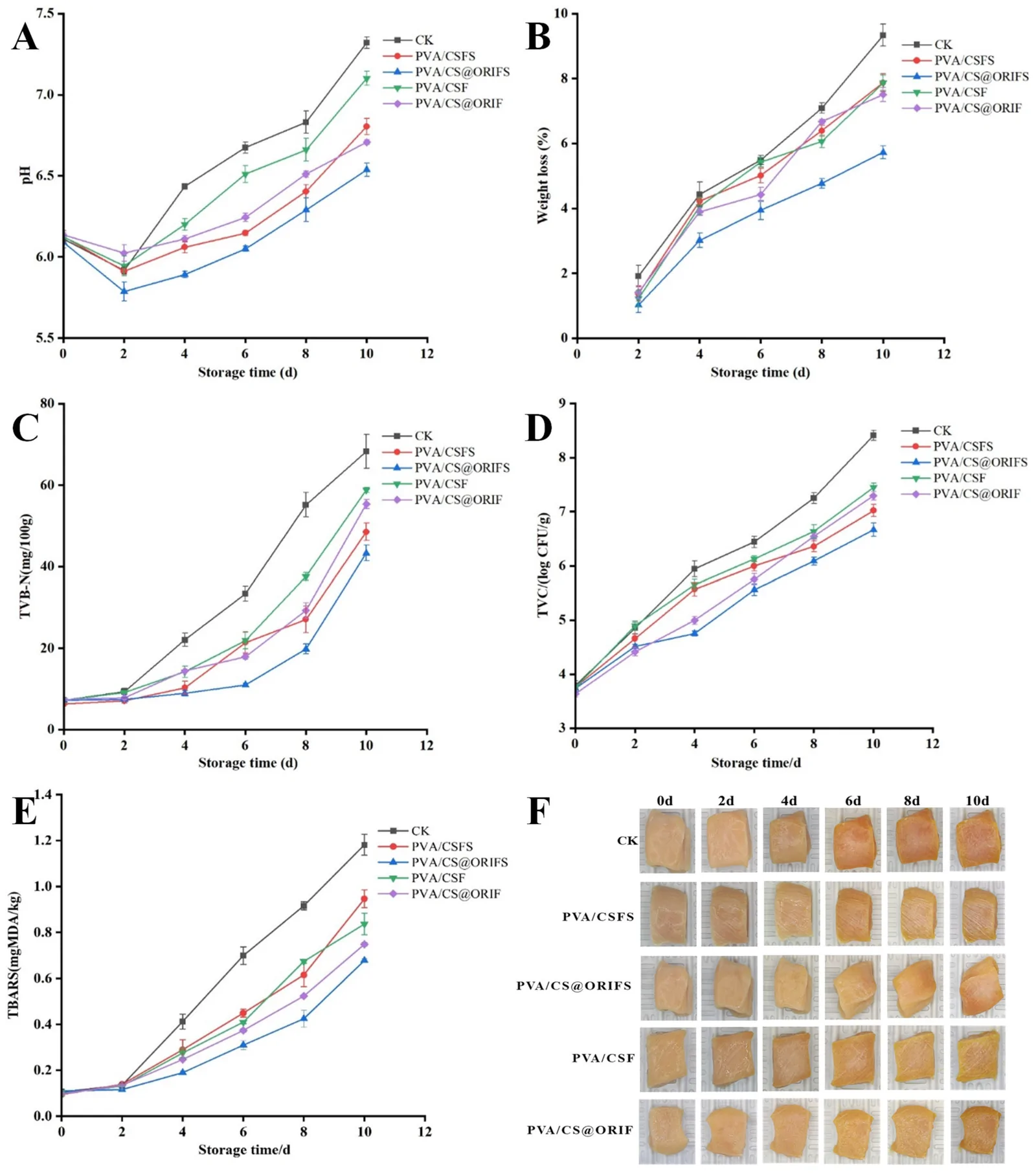
Changes in pH value (**A**), weight loss (**B**), TVB-N (**C**), TVC (**D**), TBARS (**E**) of chicken breast and changes in photographs value of meat samples (**F**) during 4 °C storage.

The color and appearance features of meat products are considered the most significant factors affecting their marketability [59]. The variations in color parameters, including a* (redness), L* (brightness), and b* (yellowness), are largely ascribed to alterations in the relative contents of oxygenated myoglobin (bright red), high iron myoglobin (grayish brown), and myoglobin (dark red), as illustrated in Figure 5F and Table 3. On day 0, all chicken breast samples appeared very fresh, with an L* value equal to 55.0. The value of L* for the control sample reduced substantially (*p* < 0.05) during storage, reaching its lowest value of 41.85 ± 0.78 on day 10. This decline in L* value resulted from lipid oxidation, which reduced meat glossiness. Relative to the control group, the PVA/CS@ORIFS and PVA/CS@ORIF treatment groups exhibited higher L* values, demonstrating that the ORI-incorporated active films inhibited the oxidation of ferrous ions to ferric ions and suppressed oxidative reactions [36].

The a* sample values in each group progressively declined, as oxygen bound with myoglobin in fresh chicken breast to form oxymyoglobin. Subsequent lipid oxidation of oxymyoglobin produced metmyoglobin, thereby shifting the color toward brown [60]. After 10 days of storage, the a* value significantly decreased (*p* < 0.05) from −1.73 ± 0.10 to −3.97 ± 0.35. The loss of red color was ascribed to the ability of microbial growth to promote the oxidation of myoglobin into metmyoglobin [61]. The data in Table 3 reveal that the PVA/CS-based coatings, whether loaded with ORI or not, were effective in delaying the discoloration of chicken breast meat. At day 10, the PVA/CS@ORIFS-coated samples showed an a* value of −2.91 ± 0.44, which was higher than that of the control. This outcome confirms that the ORI-containing composite film possesses a red color-stabilizing function, and the presence of ORI appeared to reinforce this protective action. With respect to the b* value, which serves as a marker of oxidative deterioration, no consistent variation was observed as storage progressed. For example, the PVA/CS@ORIFS group decreased from approximately 3.39 to 2.56, whereas the control group increased over storage. The irregular fluctuation in b* value readings is most likely a consequence of the randomized sampling strategy adopted during the color evaluation. This finding is consistent with observations reported in a number of earlier publications [36,62].

#### 3.5.2. pH Value

The pH value is widely used as a freshness indicator for chicken breast meat, with thresholds defined as 5.8–6.2 (fresh), 6.3–6.6 (sub-fresh), and >6.7 (spoiled) [63]. Figure 5A reveals a biphasic pH trajectory for both coated and control groups during storage—an initial decline attributed to glycolytic lactate production from glycogen breakdown [64,65], followed by a later rise driven by the gradual accumulation of alkaline metabolites (e.g., biogenic amines and ammonia) from microbial and enzymatic activities [66]. At day 6, the control (6.67) and PVA/CSF-coated (6.51) samples exhibited significantly higher pH values relative to the other groups. By day 10, the control pH had climbed to 7.32, with the most rapid increase occurring between days 6 and 10, indicating the onset of spoilage. The elevated pH value observed in the CK group may be related to the increased moisture content in the meat samples. It is plausible that this higher water activity promotes microbial proliferation and consequently generates greater quantities of alkaline compounds [67]. A slower increase in pH was observed for the antimicrobial film-treated group, possibly associated with the film effectively absorbing meat fluid and suppressing microbial growth, thus reducing meat spoilage. Notably, even on day 10, samples treated with PVA/CS@ORIFS remained at pH 6.53, indicating that they were still in a sub-fresh state. This accelerated pH increase correlated with higher moisture retention, which facilitated microbial activity and alkaline compound production [67]. In contrast, the antimicrobial film application effectively moderated pH elevation via fluid absorption and microbial suppression.

#### 3.5.3. Weight Loss Rate

As an essential quality attribute of both fresh and processed meats, water holding capacity (WHC) describes the extent to which meat retains its original or added water content during subsequent handling, processing, and storage. WHC influences visual appeal, tenderness, cooking yield, weight loss, and sensory characteristics upon consumption [68]. The main factors contributing to weight loss during meat sample preservation are water vapor diffusion and juice runoff. For the blank control group (Figure 5B), the weight loss rate exhibited a gradual increase with prolonged storage; however, the PVA/CS@ORIFS treatment group maintained substantially lower values, possibly owing to the enhanced water-binding capacity of PVA/CS films, which may help retain moisture and form an effective water barrier. Relative to PVA/CS film alone, ORI-incorporated films resulted in lower weight loss of chicken breasts throughout the 10-day storage at 4 °C, likely owing to the lipophilic nature of ORI, which contributed to the further reduction in water permeability. In conclusion, using the PVA/CS@ORI film-forming solution for preserving chilled chicken breast can provide more favorable selling conditions and a reduced juice outflow rate over a short storage period.

#### 3.5.4. TVB-N Value

The proteolytic degradation that occurs during meat storage is primarily driven by microbial activity and enzymatic reactions. This biochemical transformation generates volatile nitrogenous compounds, such as ammonia and a range of amines. Notably, these volatile basic nitrogen compounds serve as crucial indicators of meat freshness [32]. A threshold of 25 mg/100 g has been adopted in some of the literature as the upper acceptability limit for edible poultry meat products [69]. As shown in Figure 5C, control TVB-N content increased from 7.27 ± 0.32 to 22.03 ± 1.61 mg/100 g on day 4, and ultimately reached 33.37 ± 1.87 mg/100 g on day 6. By contrast, PVA/CS@ORIFS-treated samples maintained TVB-N values below the 25 mg/100 g acceptability limit for up to 8 days of storage (19.8 ± 1.21 mg/100 g). Meanwhile, samples treated with PVA/CS@ORIF (17.93 ± 0.68 mg/100 g) remained within the limit until day 6. Therefore, the results indicated that the chicken breast coated with PVA/CS@ORIFS had a favorable impact on suppressing microorganisms and successfully avoiding meat spoilage.

#### 3.5.5. TVC Value

The microbial count shows the level of spoilage and is a reliable indicator of the food’s hygienic condition. Chinese legislation has set a maximum limit of 1.0 × 10^6^ and 5.0 × 10^5^ CFU/g for fresh and frozen poultry, respectively. A threshold equal to 6 log CFU/g was adopted as the spoilage limit for meat, with values exceeding this level indicating spoilage [70]. TVC values of the specimens are shown in Figure 5D. At the start of storage, the initial microbial loads of all treatment groups were comparable, at approximately 3.70 log CFU/g. By day 4, the control TVC had escalated to 5.94 ± 0.14 log CFU/g, approaching the permissible limit. The PVA/CS-coated samples, however, exhibited TVC values below 6 log CFU/g at the same time point, suggesting that the composite film’s water absorption properties may have helped restrict microbial growth in the meat. On day 8, the PVA/CS and PVA/CSF coating samples had a TVC of 6.63 ± 0.12 and 6.35 ± 0.10 log CFU/g, respectively, crossing the threshold value. However, the PVA/CS@ORIFS specimens failed to exceed 6 log CFU/g. The released ORI from the film, together with the water absorption capacity of PVA, may have contributed to inhibiting bacterial growth. These findings demonstrate that adding ORI to the PVA/CS matrix markedly improved antibacterial performance compared with PVA/CS. When applied to chicken breast samples, the ORI-containing coating effectively inhibited bacterial proliferation.

#### 3.5.6. TBARS Value

TBARS was used to quantify MDA, a secondary oxidation product derived from unsaturated fatty acid breakdown. According to the literature, the acceptable TBA limit for meat is 0.1 mg MDA/kg, while rancid odor typically becomes detectable at 0.5–1.0 mg MDA/kg [69]. The elevation of TBARS levels is attributed to the gradual buildup of secondary lipid oxidation products (especially aldehydes). Excessive TBARS concentrations may impart objectionable flavor characteristics and off-odors to meat products. As illustrated in Figure 5E, all experimental groups displayed a continuous rise in TBARS values as storage progressed. At day 0, values were approximately 0.10 mg MDA/kg for all samples. However, with prolonged storage, the control group displayed a significantly more rapid elevation, attaining 1.18 mg MDA/kg by day10. During refrigeration, the TBARS levels of the PVA/CSF and PVA/CSFS treatment groups increased gradually, which may be linked to the antioxidant properties of CS. The beneficial role of chitosan in meat preservation has been attributed to its moderate antioxidant capacity and oxygen barrier characteristics, as reported in previous studies [68]. Chitosan coating acts as a physical shield that minimizes oxygen transfer from the ambient air to the meat surface. This oxygen-excluding function effectively impedes the progression of lipid peroxidation in the product. During refrigerated storage, the ORI incorporation into the PVA/CS solution was more successful in preventing lipid oxidation in chicken breasts than the PVA/CS coating alone. During the first four days of storage, the PVA/CS@ORIFS treatment group showed considerably reduced TBARS levels in contrast to the control group. On the 6th day, the TBARS content in the blank control group reached 0.699 ± 0.038 mg MDA/kg. In contrast, samples treated with PVA/CS@ORIFS and PVA/CS@ORIF maintained much lower values, at only 0.310 ± 0.019 and 0.374 ± 0.007 mg MDA/kg, respectively. Among all treatment groups in this study, PVA/CS@ORIFS showed the greatest inhibition of lipid oxidation, as indicated by the lowest TBARS values, suggesting that applying this coating to chicken breast effectively suppresses lipid oxidation.

Two application modes were compared in this study: a pre-formed film (PVA/CS@ORIF) and a film-forming coating (PVA/CS@ORIFS). Comparing these two modes provides practical information on how the application method affects preservation performance. In summary, the PVA/CS@ORI film-forming coating generally showed slightly better preservation performance than the pre-formed film. This conclusion is supported by the pH, TVB-N, TVC, and TBARS results. Based on TVC values, the control group reached the threshold by day 4, whereas the PVA/CS@ORIFS group remained below this threshold until day 8, corresponding to a 4-day extension of shelf life. TVB-N data (acceptability limit: 25 mg/100 g) showed a similar trend and supported this conclusion. In the coating treatment, ORI is present in the film-forming solution and can diffuse directly to the meat surface during gelation and drying, resulting in a shorter release path. In contrast, in the pre-formed film, ORI must first migrate from the film interior to the film surface before contacting the meat. In addition, the film-forming solution can wet and conform to the meat surface more closely, reducing interfacial gaps and residual oxygen, which may further limit microbial growth and lipid oxidation. The coating also comes into direct contact with exudates, potentially improving juice absorption and reducing drip loss.

## 4. Conclusions

In this study, a novel active food packaging film with antibacterial and hydrophobic functionalities was successfully developed by incorporating the antimicrobial agent ORI into a PVA and CS complex film matrix. Incorporating ORI improved the water vapor barrier and hydrophobicity characteristics, enhanced TS, and increased EAB. FTIR, SEM, XRD, and STA results confirmed that the introduction of ORI produced no discernible changes in the molecular structure of the CS/PVA composite films, showcasing excellent compatibility based on a hydrogen-bonded system. The CS/PVA@ORI film containing 75 μg/mL ORI showed superior mechanical properties and exhibited significant antibacterial activity against *S. aureus*, *L. monocytogenes*, and *E. coli*. Shelf-life evaluation further demonstrated that the film and coating containing 75 μg/mL ORI successfully inhibited the rise in pH, TVB-N content, TVC, and juice loss rate of chilled chicken breast during storage. Regarding the application method, the PVA/CS@ORI film-forming solution coating generally showed slightly better preservation performance than the pre-formed film and extended the shelf life of chilled chicken breast by 4 days. These findings highlight its potential as an active food packaging material. In subsequent studies, the safety and migration behavior of ORI, CS and PVA from films or coatings into food should be evaluated.

## Figures and Tables

**Table 1:** Physical properties of composite films with varying mass fraction of oridonin.

Physical Properties	Oridonin/µg·mL^−1^				
	0	25	50	75	100
Thickness/mm	0.039 ± 0.001 ^a^	0.039 ± 0.002 ^a^	0.040 ± 0.002 ^a^	0.039 ± 0.002 ^a^	0.039 ± 0.001 ^a^
Light transmittance/%	95.73 ± 0.40 ^a^	94.67 ± 0.55 ^ab^	93.93 ± 0.35 ^b^	91.43 ± 0.75 ^c^	90.87 ± 0.85 ^c^
TS/MPa	26.15 ± 2.06 ^bc^	23.33 ± 1.55 ^c^	28.23 ± 3.50 ^a^	31.30 ± 1.76 ^a^	27.22 ± 3.35 ^c^
EAB/%	105.13 ± 14.68 ^b^	123.38 ± 13.31 ^b^	146.32 ± 17.77 ^a^	165.88 ± 12.23 ^a^	106.82 ± 8.75 ^b^
MC/%	23.08 ± 1.24 ^a^	24.25 ± 0.56 ^a^	24.11 ± 1.71 ^a^	24.02 ± 1.86 ^a^	23.66 ± 1.25 ^a^
WA/%	995.89 ± 2.36 ^a^	882.51 ± 2.46 ^b^	845.63 ± 2.72 ^c^	749.26 ± 1.96 ^d^	625.33 ± 2.51 ^e^
WS/%	91.75 ± 3.62 ^a^	91.73 ± 2.35 ^ab^	91.43 ± 0.66 ^b^	91.10 ± 0.80 ^bc^	88.57 ± 0.54 ^c^
WVP × 10^−9^/g·mm·m^−2^·s^−1^·Pa^−1^	12.99 ± 0.70 ^a^	12.24 ± 0.27 ^b^	11.91 ± 0.32 ^bc^	11.32 ± 0.56 ^c^	11.93 ± 0.12 ^bc^

Notes: Values were reported as mean ± SD based on three separate experiments. One-way ANOVA combined with Duncan’s multiple range test was adopted to evaluate the differences among groups. a–e: Different letters within the same row indicate significant differences at *p*  < 0.05, while those with similar letters are not.

**Table 2:** Inhibition zone diameters of the composite films against *S.aureus*, *L. monocytogenes* and *E. coli.*

Oridonin/μg·mL^−1^	Inhibition Zone Diameters/mm		
	*S. aureus*	*L. monocytogenes*	*E. coli*
0	12.30 ± 0.04 ^e^	12.27 ± 0.03 ^e^	13.38 ± 0.03 ^e^
25	14.52 ± 0.04 ^d^	14.74 ± 0.04 ^d^	14.93 ± 0.02 ^d^
50	17.31 ± 0.09 ^c^	16.62 ± 0.04 ^c^	15.82 ± 0.04 ^c^
75	19.51 ± 0.05 ^b^	18.64 ± 0.03 ^b^	16.36 ± 0.04 ^b^
100	20.47 ± 0.04 ^a^	20.23 ± 0.05 ^a^	17.98 ± 0.08 ^a^

Notes: Values were reported as mean ± SD based on three separate experiments. One-way ANOVA combined with Duncan’s multiple range test was adopted to evaluate the differences among groups. a–e: Different letters within the same column indicate significant differences at *p* < 0.05.

**Table 3:** Color parameters of wrapped chicken breast samples with fabricated films during storage time at 4 °C.

Color Factors	Samples	Storage Time (Days)				
		2	4	6	8	10
L*	Control	52.37 ± 0.67 ^abA^	50.70 ± 0.85 ^abB^	46.38 ± 0.57 ^bC^	44.27 ± 0.82 ^bD^	41.85 ± 0.78 ^dE^
	PVA/CSFS	53.22 ± 0.30 ^aA^	51.63 ± 0.47 ^aB^	50.54 ± 0.92 ^aB^	49.07 ± 0.83 ^aC^	43.21 ± 0.35 ^cD^
	PVA/CS@ORIFS	52.13 ± 0.71 ^abA^	51.48 ± 0.61 ^abAB^	50.83 ± 0.17 ^aB^	49.21 ± 0.32 ^aC^	47.14 ± 0.64 ^aD^
	PVA/CSF	51.79 ± 0.81 ^bA^	50.07 ± 0.59 ^bB^	49.66 ± 0.75 ^aB^	47.92 ± 0.37 ^aC^	45.70 ± 0.53 ^bD^
	PVA/CS@ORIF	52.20 ± 0.19 ^abA^	51.15 ± 0.8 ^abA^	49.88 ± 0.32 ^aB^	48.75 ± 0.94 ^aB^	45.89 ± 069 ^bC^
a*	Control	−1.73 ± 0.10 ^aA^	−2.49 ± 0.19 ^bB^	−2.71 ± 0.16 ^bC^	−3.19 ± 0.25 ^bD^	−3.97 ± 0.35 ^bE^
	PVA/CSFS	−1.76 ± 0.23 ^aA^	−1.94 ± 0.21 ^aAB^	−2.33 ± 0.16 ^abBC^	−2.58 ± 0.21 ^aCD^	−2.95 ± 0.23 ^aD^
	PVA/CS@ORIFS	−1.71 ± 0.08 ^aA^	−1.84 ± 0.18 ^aA^	−1.95 ± 0.08 ^aAB^	−2.4 ± 0.15 ^aBC^	−2.91 ± 0.44 ^aC^
	PVA/CSF	−1.73 ± 0.26 ^aA^	−1.91 ± 0.07 ^aAB^	−2.16 ± 0.13 ^abBC^	−2.49 ± 0.16 ^abC^	−3.35 ± 0.23 ^abD^
	PVA/CS@ORIF	−1.72 ± 0.25 ^aA^	−1.82 ± 0.23 ^aAB^	−2.09 ± 0.26 ^abAB^	−2.37 ± 0.29 ^aCD^	−2.95 ± 0.42 ^aD^
b*	Control	2.17 ± 0.89 ^bB^	2.40 ± 0.17 ^bB^	3.33 ± 0.47 ^bcAB^	4.67 ± 0.75 ^aAB^	5.79 ± 2.62 ^aA^
	PVA/CSFS	3.07 ± 0.87 ^abB^	3.47 ± 0.79 ^bB^	4.82 ± 0.85 ^aA^	3.88 ± 0.41 ^aAB^	4.32 ± 0.28 ^abB^
	PVA/CS@ORIFS	3.39 ± 0.75 ^abAB^	3.31 ± 0.58 ^bAB^	3.71 ± 0.41 ^bcA^	4.40 ± 0.75 ^aA^	2.56 ± 0.12 ^bB^
	PVA/CSF	3.41 ± 0.27 ^abB^	3.51 ± 0.08 ^bB^	3.91 ± 0.44 ^abAB^	4.31 ± 0.55 ^aAB^	5.06 ± 1.15 ^abA^
	PVA/CS@ORIF	4.43 ± 0.58 ^aA^	5.82 ± 0.82 ^aB^	2.86 ± 0.20 ^cB^	3.98 ± 0.65 ^aC^	4.00 ± 0.46 ^abB^

Notes: Data are presented as mean ± SD based on three independent experiments. For the chicken breast storage experiment, one-way ANOVA followed by Duncan’s multiple range test was performed separately at each storage time point to compare the effects of different packaging treatments (CK, PVA/CSFS, PVA/CS@ORIFS, PVA/CSF, and PVA/CS@ORIF), and separately within each packaging treatment to compare the effects of storage time (2, 4, 6, 8, and 10 days). A–E: Values with different capital letters in the same row within the same packaging type were significantly different at *p* < 0.05. a–d: Values with different lowercase letters in the same column within the same storage time were significantly different at *p* < 0.05.

## Data Availability

The original contributions presented in this study are included in the article. Further inquiries can be directed to the corresponding author.
